# Effect of a probiotic and an antibiotic on the mobilome of the porcine microbiota

**DOI:** 10.3389/fgene.2024.1355134

**Published:** 2024-03-28

**Authors:** Xavier C. Monger, Linda Saucier, Frédéric Guay, Annie Turcotte, Joanie Lemieux, Eric Pouliot, Sylvain Fournaise, Antony T. Vincent

**Affiliations:** ^1^ Département des Sciences Animales, Université Laval, Québec, QC, Canada; ^2^ Institut de Biologie Intégrative et des Systèmes, Université Laval, Québec, QC, Canada; ^3^ Institut sur La Nutrition et Les Aliments Fonctionnels, Université Laval, Québec, QC, Canada; ^4^ Centre de Recherche en Infectiologie Porcine et Avicole, Faculté de Médecine Vétérinaire, Université de Montréal, Saint-Hyacinthe, QC, Canada; ^5^ Département de Biologie, Microbiologie, Université de Sherbrooke, Sherbrooke, QC, Canada; ^6^ Département de Biochimie, Microbiologie et Bio-informatique, Université Laval, Québec, QC, Canada; ^7^ Olymel S.E.C./L.P., Boucherville, QC, Canada

**Keywords:** antibiotics resistance, digesta, faeces, gut microbiome, metagenomics, plasmids, swine

## Abstract

**Introduction:** To consider the growing health issues caused by antibiotic resistance from a “one health” perspective, the contribution of meat production needs to be addressed. While antibiotic resistance is naturally present in microbial communities, the treatment of farm animals with antibiotics causes an increase in antibiotic resistance genes (ARG) in the gut microbiome. Pigs are among the most prevalent animals in agriculture; therefore, reducing the prevalence of antibiotic-resistant bacteria in the pig gut microbiome could reduce the spread of antibiotic resistance. Probiotics are often studied as a way to modulate the microbiome and are, therefore, an interesting way to potentially decrease antibiotic resistance.

**Methods:** To assess the efficacy of a probiotic to reduce the prevalence of ARGs in the pig microbiome, six pigs received either treatment with antibiotics (tylvalosin), probiotics (*Pediococcus acidilactici* MA18/5M; Biopower^®^ PA), or a combination of both. Their faeces and ileal digesta were collected and DNA was extracted for whole genome shotgun sequencing. The reads were compared with taxonomy and ARG databases to identify the taxa and resistance genes in the samples.

**Results:** The results showed that the ARG profiles in the faeces of the antibiotic and combination treatments were similar, and both were different from the profiles of the probiotic treatment (*p* < 0.05). The effects of the treatments were different in the digesta and faeces. Many macrolide resistance genes were detected in a higher proportion in the microbiome of the pigs treated with antibiotics or the combination of probiotics and antibiotics. Resistance-carrying conjugative plasmids and horizontal transfer genes were also amplified in faeces samples for the antibiotic and combined treatments. There was no effect of treatment on the short chain fatty acid content in the digesta or the faeces.

**Conclusion:** There is no positive effect of adding probiotics to an antibiotic treatment when these treatments are administered simultaneously.

## 1 Introduction

Antibiotic resistance has been a major health concern worldwide for many years, and yet it is still a growing problem in human and animal health (WHO, 2020). While resistance to antibiotics predates their discovery, the selective pressure from antibiotic use, notably in medicine and agriculture, is contributing to the rise of resistant or multi-resistant bacteria (Larkin, 2023). This phenomenon is exacerbated by the capacity of bacteria to evolve rapidly and exchange genetic material between species (Khan and Rao, 2019; Qu and Chen, 2022). When resistance is acquired by pathogenic bacteria, it threatens the efficacy of antibiotic treatments against the diseases caused by the bacteria (Pan et al., 2022).

Antibiotics began to be used in agriculture shortly after their discovery, first to treat and prevent disease in animals and then as growth promoters; however, the latter purpose is no longer socially accepted and no longer used in many countries (OIE, 2018). With the growing awareness of antibiotic use on the selection of resistance, there is an increasing will to further reduce antibiotic use; however, there is still a need to treat sick animals. Animals are particularly vulnerable to infections during the post-weaning stage, and antibiotics are still commonly used to prevent mortality during this period (Canibe et al., 2022). The frequency of treatments and the large number of animals being raised makes pig production particularly interesting to investigate when it comes to the agricultural use of antibiotics (Monger et al., 2021).

Pig farms can contribute to the spread of antimicrobial resistance in many ways. The selection of resistant bacteria and the horizontal transfer of resistance genes originates in the gut microbiota of the animals after they are treated with antibiotics, with this effect being even stronger after oral treatment than after injection (Ricker et al., 2020). Microorganisms can be transferred to the environment through air, manure, wastewater, and occasionally through the meat (Monger et al., 2021).

Many alternatives to antibiotics have been explored in pig rearing, including pre- and probiotics and essential oils (Lopez-Galvez et al., 2021; Pereira et al., 2022). Probiotics are often studied to improve microbiome health in agriculture, and show potential to not only reduce the use of antibiotics in disease prevention but also as a growth promoter (Joysowal et al., 2021; Pereira et al., 2022; Pezsa et al., 2022; Guevarra et al., 2023). Although their effects on growth, feed efficiency, and resistance to infections has been studied to some extent, the mechanisms by which they achieve results and their complex interactions with the animal microbiota are still not fully understood. The interactions between probiotics and antibiotics are also not well understood, although they can reduce the population of pathogenic bacteria, by competition (Shin et al., 2019) and production of bacteriocin (Kim et al., 2023). Whether or not this effect could reduce the acquisition of resistance to antibiotics in pathogens is still unknown.

The effect of interaction between antibiotics, probiotics, and the pig gut microbiome on antibiotics resistance was investigated in this study. To accomplish this, pigs were administered probiotics, antibiotics, and a combination of both. Their faeces and digesta were collected and the DNA from those samples was extracted and sequenced. The sequences were then analyzed to give insight into how the gut microbiome of the pigs was affected by antibiotic or probiotic treatment at the genomic level.

## 2 Materials and methods

### 2.1 Animal housing and care

**FIGURE 1:**
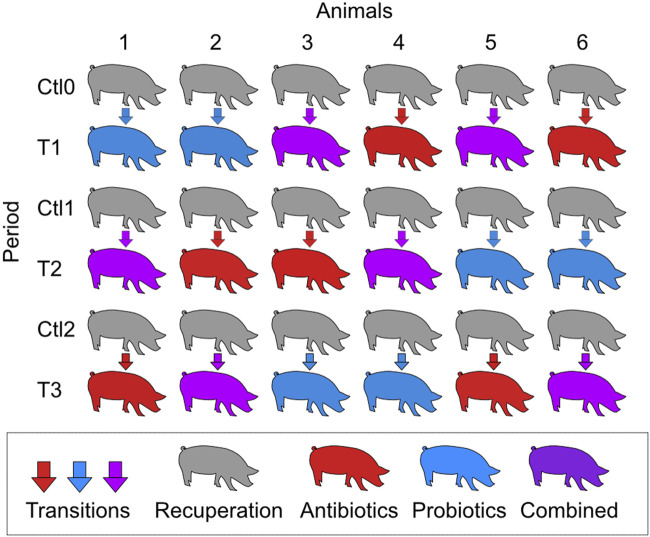
Schematic representation of the crossover experimental design. T1, T2, and T3 refer to the treatment periods. The treatments were a macrolide antibiotic (tylvalosin; 250 g/ton of feed), a commercial probiotic (*Pediococcus acidilactici* MA18/5M, 10^8^ CFUs/day), and a combination of both treatments at the same concentrations as the individual treatments. During the control periods (Ctl0, Ctl1, and Ctl2), the animals were fed the same diet without treatment. Each period lasted 3 weeks.

Animal housing conditions were described in a previously published paper (Monger et al., 2022). The experimental design was approved by the Animal Protection Committee of Université Laval prior to the experiments (2019057–1). Briefly, six Yorkshire-Landrace male pigs at 55 days of age were transported to the Université Laval animal research facility. At 66 days, they underwent surgery to implant an ileal-T canula, as previously described (Wubben et al., 2001). They were allowed a 11-day recovery period before the beginning of the treatments, as it was demonstrated that inflammation at the cannulation site was negligible 7 days after the surgery (Zhang et al., 2014). The six pigs received three different treatments: macrolide antibiotic (tylvalosin TYL; 250 g/ton of feed; Aivlosin^®^ (17% tylvalosin), ECO Animal Health Princeton, NJ, USA), probiotic *Pediococcus acidilactici* MA18/5M (PA; 10^8^ CFUs/day; Biopower^®^ PA, Lallemand Animal Nutrition, Milwaukee, WI, USA), and a combination of both antibiotic and probiotic treatment at the same concentrations as the individual treatments. Each pig received the three treatments in a different order, and a period of recovery was given between each treatment with a control diet not supplemented, according to a crossover design (Figure 1). The periods of treatment and recovery were 3 weeks each.

### 2.2 Sample collection and processing

Digesta and faeces from each pig were collected at the end of each treatment period for a total of 36 faeces samples and 35 digesta samples. The digesta from pig number two at the Ctl2 period could not be collected as there was no digesta at the time of sampling. All the samples were stabilized using the PERFORMAbiome•GUT | PB-200 sampling kit according to the manufacturer’s instructions (DNAgenotek, Ottawa, Ontario, Canada) and as suggested elsewhere (Monger et al., 2022).

The DNA was extracted from the samples with the QIAamp PowerFecal Pro DNA Kit (QIAGEN, Toronto, Canada) using 250 µL of stabilized faeces or digesta. The DNA was quantified using the PicoGreen kit (Invitrogen, Waltham, Massachusetts, USA). Integrity of the DNA was assessed by electrophoresis on an agarose gel.

Short chain fatty acids (SCFAs) were quantified as previously described (Laforge et al., 2023). Briefly, samples were stored at −80°C, then thawed and homogenized in water. The homogenized samples were centrifugated and SCFAs were extracted by liquid-liquid extraction. The SCFAs were subjected to gas chromatography coupled with a flame ionization detector (GC-FID Shimadzu, Kyoto, Japan) following the protocol published by Roussel et al. (2022).

### 2.3 Sequencing and data analysis

Shotgun sequencing libraries were generated from 50 ng of gDNA using the NEBNext Ultra II DNA Library Prep Kit for Illumina (New England BioLabs, Whitby, Canada) as per the manufacturer’s recommendations. Sequencing was done on an Illumina NovaSeq 6000 by the Genome Quebec Centre of Expertise and Services (Montréal, Canada). Base calling was performed with Illumina’s Real Time Analysis software version 3.4.4. A minimum of 70 million paired reads was generated for each of the samples. The optical duplicates in the reads were removed with the clumpify tool from bbmap version 38.96 (Bushnell, 2014). The reads were then filtered using fastp version 0.23.1 (Chen S. et al., 2018), and the sequences were mapped on pig genome sequences using bowtie2 version 2.4.4 (Langmead and Salzberg, 2012) and samtools version 1.17 (Li et al., 2009) to keep only those of the microbiome. The reference genome used was Sscrofa11.1 (RefSeq: GCF_000003025.6). The number of reads at each step is presented in Supplementary Table S1. The whole genome shotgun sequencing data set was deposited in the NCBI Sequence Read Archive database under the BioProject ID PRJNA1049315.

The reads were then analyzed and coassembled with the SqueezeMeta pipeline, version 1.5.1, using default parameters (Tamames and Puente-Sanchez, 2019). Only contigs with a length greater than or equal to 200 bp were retained for further analysis. The SQMtools R package (Puente-Sanchez et al., 2020) was used to import the data in R. The vegan R package (Dixon, 2003) was used for alpha diversity analysis, and the phyloseq (McMurdie and Holmes, 2013) and microbial (Kai, 2021) packages were used for beta diversity and linear discriminant analysis (LDA) analysis, respectively. The cutoffs for LDA were a score greater than or equal to two and an adjusted *p*-value less than 0.05. The pvclust package was used for hierarchical clustering of the data with 10,000 bootstrap replications (Suzuki and Shimodaira, 2006).

The assembly was screened for plasmidic contigs using PlasForest version 1.4 (Pradier et al., 2021). The plasmid sequences were reconstructed from contigs using the MOB-suite version 3.1.4 (Robertson and Nash, 2018). ARGs were identified by mapping the reads against the Comprehensive Antibiotic Resistance Database (CARD) (Alcock et al., 2020) using MetaProtMiner with a minimum similarity of 90% and a query cover of 80% (Galiot et al., 2023).

The 16S rRNA gene was sequenced and analyzed as described elsewhere (Laforge et al., 2023). Briefly, the v3–v4 region of the 16S DNA was sequenced at the Plateforme d’analyse génomique of the Institut de Biologie Intégrative et des Systèmes (Université Laval, Quebec City, Canada). The sequences were pretreated and taxonomically assigned with the R package DADA2 (Callahan et al., 2016). During filtration, the first 17 nucleotides of the forward reads and the first 21 nucleotides of the reverse reads were trimmed to remove primers. Dereplication, sample inference, chimera identification, and merging of the paired-end reads were performed using the default parameters. Taxonomic assignment was conducted using the SILVA rRNA database (release 138.1) with the naive Bayesian classifier method (utilizing the assignTaxonomy command of the DADA2 package). The sequencing data set was deposited in the NCBI Sequence Read Archive database under the BioProject ID PRJNA1049315.

### 2.4 Statistical analysis

Statistical analyses were performed using the prism software version 9.2.0. For multiple comparisons, data normality was assessed using the Shapiro-Wilk test. A Kruskal–Wallis test followed by a *post hoc* Dunn’s test was used to analyze alpha diversity and short-chain fatty acids. A Tukey test was used to compare antibiotic resistance gene scores. Adjusted *p*-values were employed to evaluate the significance of the results. Statistical analysis of the beta diversity was performed with the adonis function from the vegan package, which is a function to compute permanova tests with 10,000 iterations. Correlation analysis of the SCFAs data (lactic, propionic, butyric, and acetic acids) with bacterial taxa was done with R using the ccrepe package and 10,000 iterations (Schwager et al., 2019). Only correlations with compositionality corrected *p*-values less than 0.05 were considered significant.

## 3 Results

### 3.1 Taxonomic profile

**FIGURE 2:**
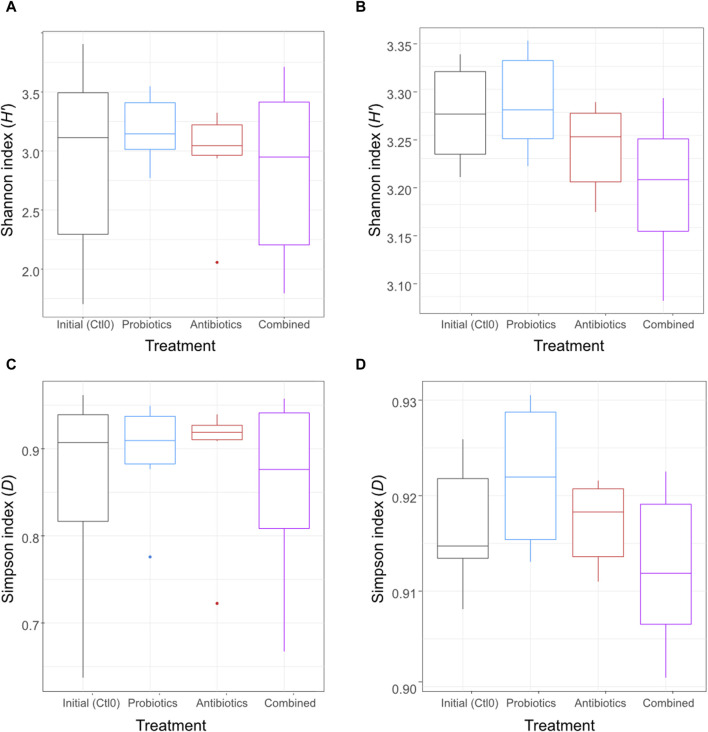
Alpha diversity using Shannon index of the digesta **(A)** and the faeces **(B)** and alpha diversity using the Simpson index of the digesta **(C)** and the faeces **(D)** for animals treated with the commercial probiotic *Pediococcus acidilactici* MA18/5M (blue), a macrolide antibiotic (tylvalosin, red), or a combination of both (purple). Data from samples taken before the animals received treatments (Ctl0) are shown in black. Box plots show means and quartiles. No comparison was significant (*p* > 0.05, Kruskal–Wallis test).

An antibiotic treatment (tylvalosin), a probiotic treatment (*P. acidilactici*), and a combination of both treatments were administered to six pigs to investigate the resilience and adaptation of their gut microbiome using metagenomic analysis of their faeces and ileal digesta samples. To assess the effect of the treatments on the microbial diversity within the samples (alpha diversity), the Shannon and Simpson diversity indexes were computed for all samples and the treatments were compared (Figure 2). Both indexes are a measure of diversity considering evenness and number of taxa; the Shannon index is more influenced by richness, and the Simpson index is more influenced by evenness of repartition and less influenced by rare species.

No significant difference was observed (*p* > 0.05) between treatments for either digesta (Figures 2A, C) or faeces (Figures 2B, D) samples. The metataxonomic analysis of 16S rRNA gene sequences did not reveal any significant difference between treatments for either sample type (Supplementary Figure S1).

**FIGURE 3:**
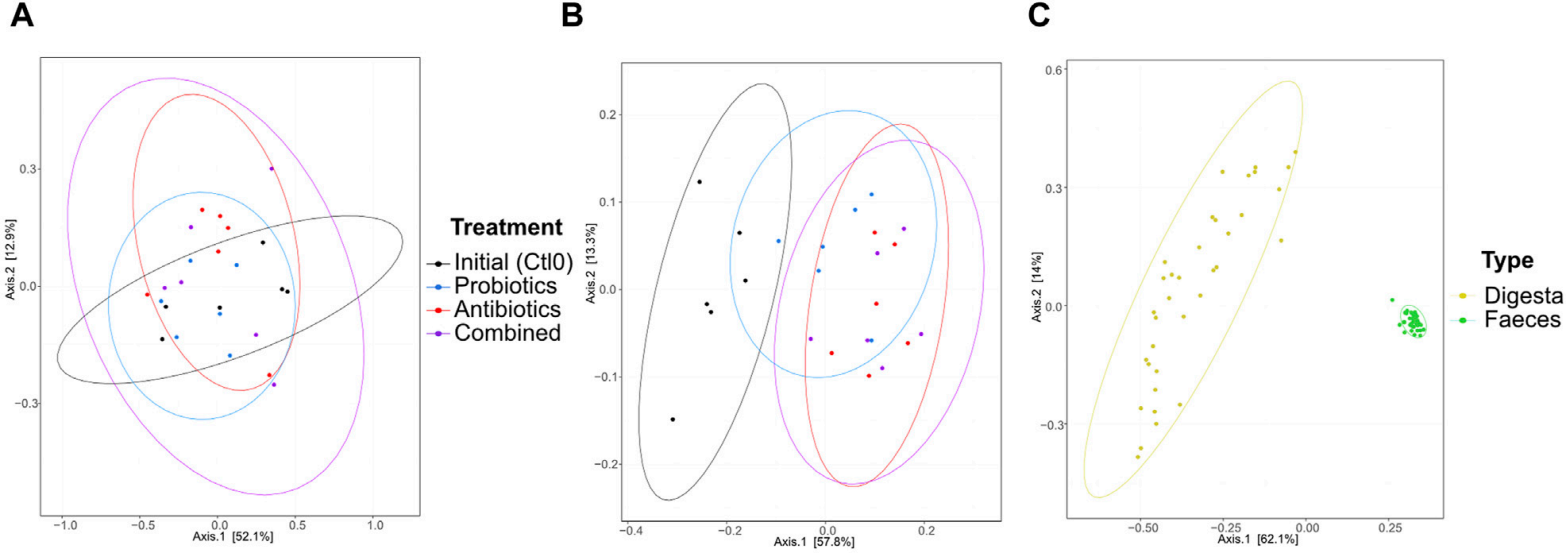
Beta diversity represented by a principal coordinate analysis plot using Bray-Curtis dissimilarities of bacterial taxa from the digesta **(A)**, the faeces **(B)**, and all samples **(C)** for animals treated with the commercial probiotic *Pediococcus acidilactici* MA18/5M (blue), a macrolide antibiotic (tylvalosin, red), or a combination of both (purple). Data from samples taken before the animals received treatments (Ctl0) are shown in black. Each point represents a sample. The distance between points reflects the difference in microbial composition between samples; closer points indicate higher similarity. The principal axes represent dimensions that maximize variance among samples. Percentages indicate the proportion of variance explained by each axis. The ellipses represent a 95% confidence interval.

The dissimilarity between the samples (beta diversity) was visualized by a principal coordinate analysis (PCoA) plot of the Bray-Curtis distance (Figure 3). The treatments had no significant effect on the microbiota composition of the digesta (*p* > 0.05; Figure 3A). In the faeces (Figure 3B), all groups were different from the first control period (all *p* < 0.0096). No other groups were found to be significantly different. As expected, the digesta and the faeces were significantly different from each other (*p* < 0.0001, Figure 3C) and the variability between faeces samples was greater than the variability between digesta samples. Also, 16S rRNA gene metagenomics validated that the microbiota remained stable between treatments (Supplementary Figure S2).

The microbiota composition is similar between treatments (Supplementary Figure S3) and as expected (Holman et al., 2017). The digesta is predominantly composed of the *Lactobacillaceae* and *Peptostreptococcaceae* families, accounting for more than half of the microbial composition. Notably, there is a significant increase in *Streptococcaceae* observed in the samples taken after probiotic treatment. The composition of the feces is more evenly distributed among various taxa, including several families of unclassified *Eubacteriales*, *Bacteroidales*, *Firmicutes*, and *Clostridia*, as well as the *Prevotellaceae* family. However, as anticipated, the fecal microbiome is initially largely colonized by bacteria from the *Prevotellaceae* family at the beginning of the experiment, followed by a subsequent reduction.

Marker taxa were identified using LDA and some of the bacteria varied significantly (*p* < 0.05) between the antibiotic and probiotic groups (Supplementary Figure S4). Interestingly, the genera that were significantly different between these two groups were only detected during treatment with the probiotic. The most amplified genus in the digesta was the *Methanosphaera* archaea, while in the faeces it was an unclassified bacterial genus belonging to the *Akkermansiaceae* family.

### 3.2 Resistome profile

**FIGURE 4:**
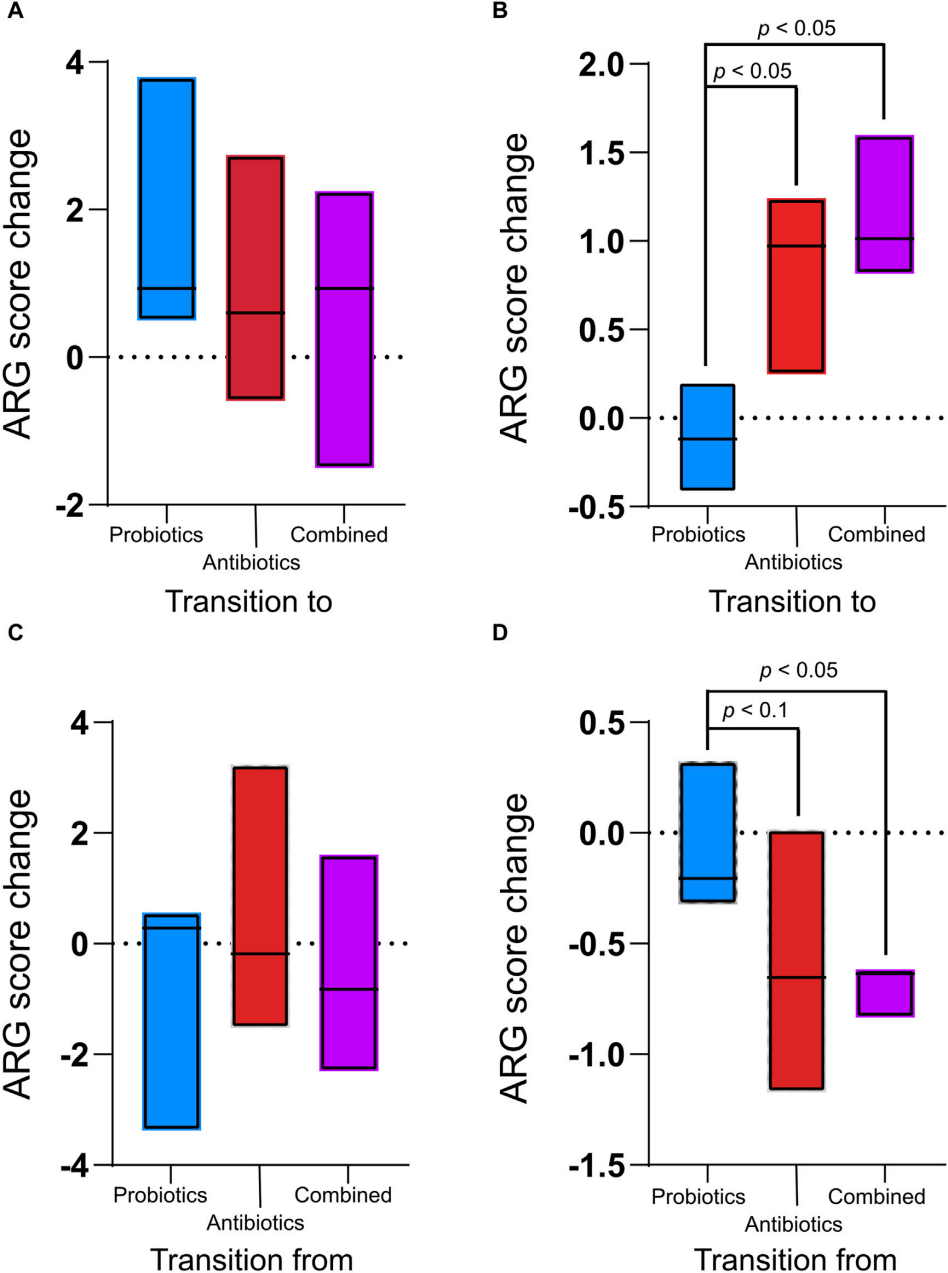
Antibiotic resistance gene (ARG) score change between the treatments (antibiotics, probiotics, or a combination of both) and their preceding recuperation period in the digesta **(A)** and the faeces **(B)** and ARG score change between recuperation periods and the following treatment period in the digesta **(C)** and the faeces **(D)**. The ARG score, computed using MetaProtMiner (Galiot et al., 2023), for a sample corresponds to the sum of reads aligned to each antibiotic resistance gene, divided by the total gene length and the number of reads for the dataset, multiplied by one million. Only *p*-values less than 0.05 (Tukey test) are shown.

For each treatment period, the ARG score (calculated by MetaProtMiner) of the previous recuperation period was subtracted from the score calculated for each treatment period to assess only the effect of the treatment (Figures 4A, B). There was no significant difference in digesta samples between treatments (*p* > 0.05, Figure 4A). However, in the faecal samples, the ARG score change caused by the treatments was significantly higher for the antibiotics and combined treatments than for the probiotic treatment (Figure 4B).

The ARG score for the preceding treatment was subtracted from the score for each recuperation period to verify the resilience of the animals’ microbiome following treatment. During the recuperation periods following treatments, the ARG score change was not significantly different between treatments for the digesta (Figure 4C). However, in the faeces, ARGs decreased during the recuperation period following the antibiotic and combined treatments (Figure 4D). The change during recuperation from the combined treatment was significantly lower than during recuperation from the probiotic treatment (*p* = 0.04). There was a tendency (*p* < 0.061) for the decrease in ARGs to be smaller during recuperation after antibiotic treatment than during recuperation from probiotic treatment. In the faeces for the combined treatment, the decrease in ARGs during recuperation was smaller than the increase during treatment (*p* = 0.02), while the difference was not significant for the antibiotic treatment (*p* = 0.31).

**FIGURE 5:**
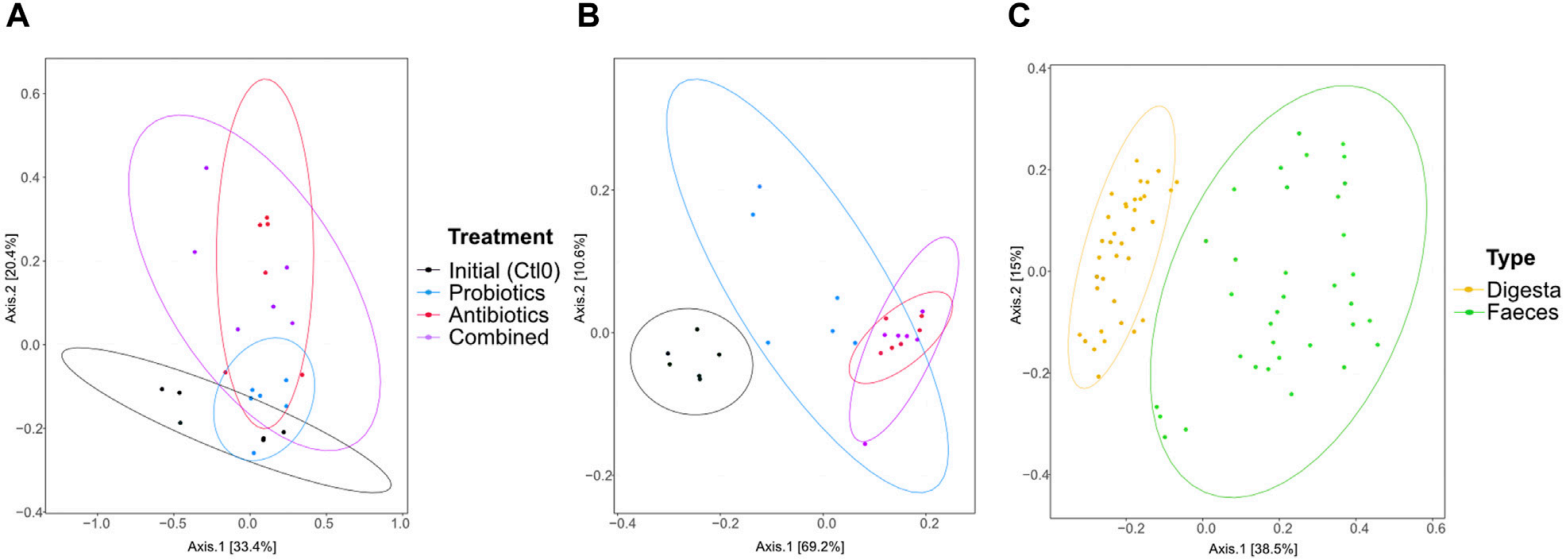
PCoA of the resistome based on the antibiotic resistance gene (ARG) scores during the first recuperation period (Ctl0) and the treatment periods (antibiotics, probiotics, or a combination of both) of the digesta **(A)**, the faeces **(B)**, and all samples **(C)**. The ARG score, computed using MetaProtMiner (Galiot et al., 2023), for a sample corresponds to the sum of reads aligned to each antibiotic resistance gene, divided by the total gene length and the number of reads for the dataset, multiplied by one million. Each point represents a sample. The distance between points reflects the difference in resistance genes composition between samples; closer points indicate higher similarity. The principal axes represent dimensions that maximize variance among samples. Percentages indicate the proportion of variance explained by each axis. The ellipses represent a 95% confidence interval.

Sample grouping based on the resistome profile was done with a PCoA plot using Bray-Curtis dissimilarity (Figure 5). In the digesta (Figure 5A), significant differences were observed between the initial microbiome (control) and the antibiotics treatment (*p* = 0.046), but the comparison was not significant between the initial microbiome and the combined treatment, although the *p*-value is close to 0.05 (*p* = 0.051). Differences were also found between the probiotic treatment and antibiotic treatment (*p* = 0.037) and the combined treatment (*p* = 0.005). In the faeces (Figure 5B), all groups were different (all *p* < 0.003) except for the combined and antibiotic treatments (*p* = 0.7534). The resistome profile was also different between the total samples coming from the digesta and the faeces (*p* < 0.0001, Figure 5C). More variability in the resistome profile was observed in the faeces samples from the probiotic treatment compared with the combined and antibiotic treatments. Interestingly, the opposite trend was observed in the digesta samples: the samples from the probiotic treatment group were less variable than the combined and antibiotic treatment groups. Globally, there was also more variability in the faeces samples than in the digesta samples (Figure 5C).

**FIGURE 6:**
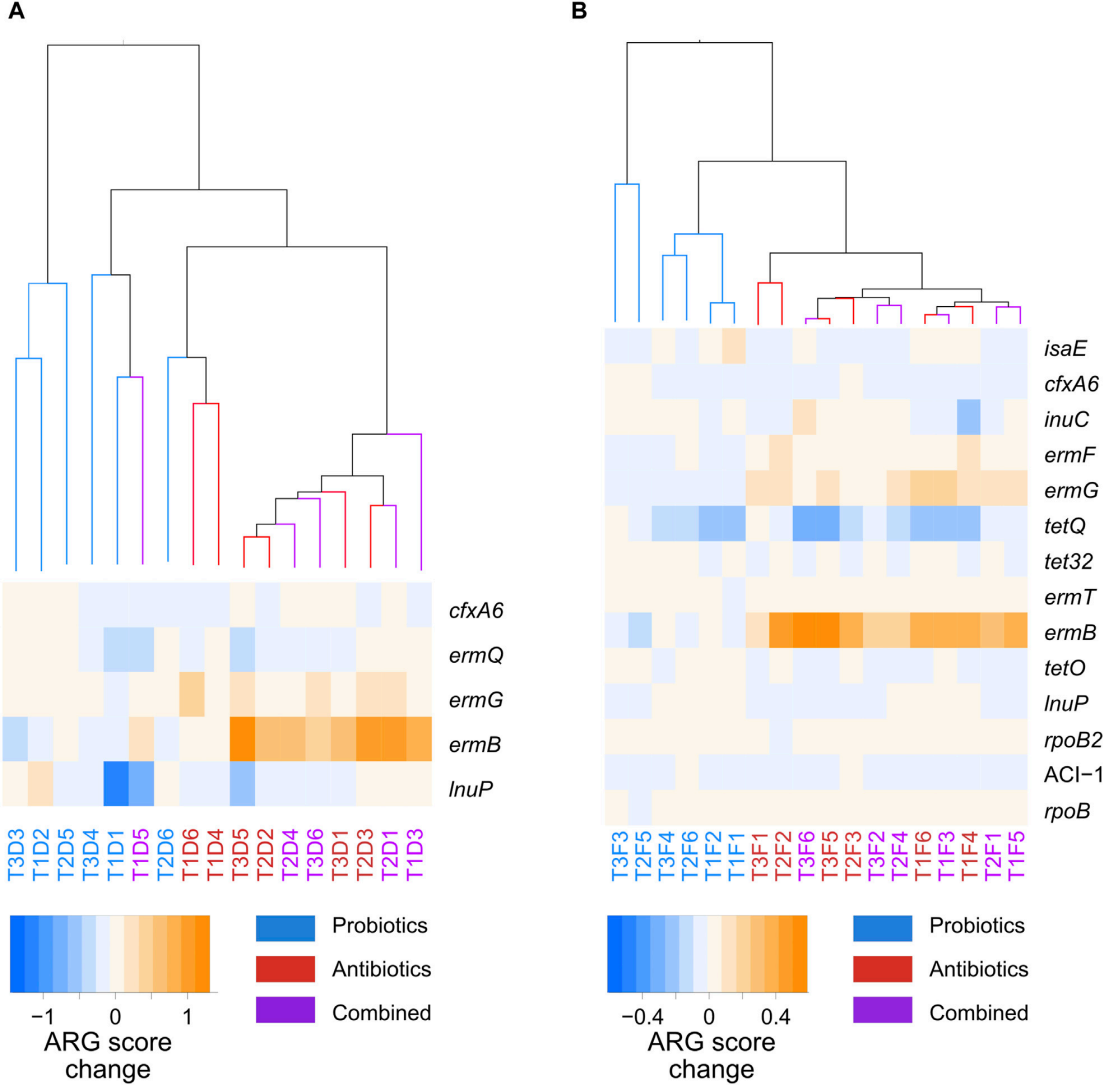
Hierarchical clustering of the whole-resistome changes caused by the treatments, and a heatmap of the marker genes between probiotic, antibiotic, and a combination of both treatments for samples taken from the digesta **(A)** and the faeces **(B)**. The samples names correspond to the treatment period of the samples: T1, T2, or T3; the nature of the samples: faeces (F) or digesta (D); and the pig from which the sample was taken: pig 1 to 6. Blue corresponds to probiotic treatment, red to antibiotic treatment, and purple to a combination of both treatments.

Marker resistance genes between the probiotic and antibiotic treatments were identified with LDA analysis (Supplementary Figure S5); no markers were found between the combined and antibiotic treatments. A heatmap of the abundance score of those genes across samples is shown in Figure 6. In both faeces and digesta, hierarchical clustering analysis of the transitions in the whole resistome between the control and treatments showed that the changes induced by the probiotic treatment clustered together, while the changes induced by the antibiotic or combined treatments clustered together (Figure 6). The ARGs that were considered markers with LDA were amplified in the transition to antibiotic and combined treatments and were stable or diminished in the transition to probiotic treatment. Five ARG markers were found in the digesta (Figure 6A), of which *ermB*, *ermG*, and *ermQ* were macrolide resistance genes. A total of 14 ARG markers were found for the faeces samples (Figure 6B), and five of these (*ermB*, *ermG*, *ermT*, *ermQ*, and *ermF*) were macrolide resistance genes. This is not surprising, as tylvalosin is an antibiotic of the macrolide class. Interestingly, all the genes identified as markers in the digesta samples were also markers in the faeces samples.

### 3.3 Plasmidome profile

After screening of the total assembly for plasmidic sequences, 38,565 of 9,444,776 contigs were identified to be putatively plasmidic. These sequences were compared with the CARD database, and 27 contigs were carrying at least one ARG and two contigs were carrying two ARGs. No contigs were found carrying more than two ARGs. Plasmid sequences were reconstructed from contigs, and those carrying ARGs are described in Table 1. Two plasmids, AA998 and AA448, were carrying multiple ARGs, and both were considered as conjugative plasmids by the tool mob_suite. The plasmid AA448 is associated with *Klebsiella pneumonia* and was carrying genes for resistance against the tetracycline, gentamycin, and sulfonamide classes of antibiotics. The plasmid AA998 was associated with *Salmonella enterica* serovar Typhi and was carrying resistance genes against the beta-lactam, tetracycline, chloramphenicol, and macrolide classes of antibiotics (Table 1).

**TABLE 1:** Description of antibiotic resistance gene–carrying plasmids found in all digesta and faeces samples.

Plasmid	Host	Predicted mobility	Number of resistance genes	Resistance genes	Size (bp)	Completion (%)	Amplified in antibiotics treatments
AA998	*Salmonella enterica* subsp. *enterica* serovar Typhi str. CT18	conjugative	7	*blaTEM-1, tet(B), cmlA1, aadA2, msr(E), mph(E), catA1*	288854	100	Yes
AA474	*Escherichia coli*	mobilizable	1	*blaCMY-2*	33953	100	Yes
AG658	*Escherichia coli*	non-mobilizable	1	*sul3*	4068	100	No
AA448	*Klebsiella pneumoniae* subsp. *pneumoniae* HS11286	conjugative	3	*tet(A), sul1, aac(3)-VIa*	133888	100	Yes
AB881	*Lactobacillus plantarum*	non-mobilizable	1	*lnu(A)*	3075	100	No
AB968	*Lactococcus lactis subsp. lactis* K214	non-mobilizable	1	*tet(S)*	7652	100	No
AB756	*Enterococcus faecium*	non-mobilizable	1	*poxtA*	11934	100	Yes

To identify marker plasmidic contigs between the antibiotic and probiotic treatments, LDA analysis was performed (Supplementary Figure S6). In the digesta, all maker plasmids were amplified in the probiotic treatment; however, none of the marker contigs were carrying ARGs (Supplementary Figure S6). In the faeces, all markers for plasmidic contigs were amplified in the antibiotic treatment. Four of the contigs that were markers in the faeces were carrying ARGs, and three of these belonged to plasmids that were either conjugative or mobilizable.

Samples were grouped based on the transitions in the whole plasmidome between the control and treatments using hierarchical clustering with heatmaps of the transition of the ARG-carrying plasmids (Supplementary Figure S7). The whole plasmidome of the samples did not cluster according to treatments.

### 3.4 Functional profile

**FIGURE 7:**
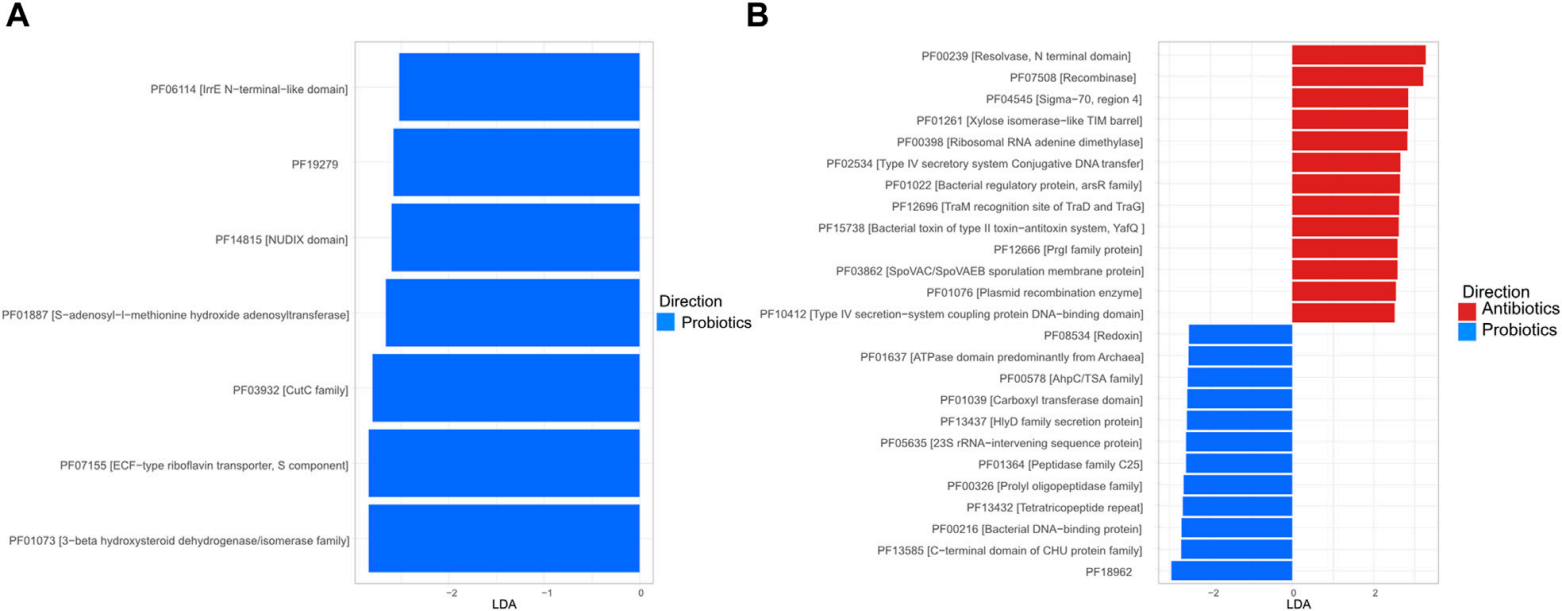
Linear discriminant analysis (LDA) plot of the marker function, as defined in the Pfam database, in the digesta **(A)** and the faeces **(B)**. For the digesta, only samples from animals fed with the probiotic had markers (score ≥2 and a *p* < 0.05).

To more specifically assess the role of the treatments on functional profile, the relative abundance of functions, as defined by the Pfam database (Finn et al., 2008), was established and markers were identified using LDA analysis. In the digesta, all the marker functions were more frequent in the probiotics group and no horizontal transfer functions were identified as markers (Figures 7A). In the faeces, two of the functions identified as markers for antibiotic treatments were recombinase (PF07508 [Recombinase]) and resolvase (PF00239 [Resolvase, N terminal domain]); these two functions play a role in gene exchange (Figures 7B) (Johnson and Grossman, 2015; Rice, 2015).

Four SCFAs were measured in the samples: acetic acid, lactic acid, propionic acid, and butyric acid; these are important metabolites for host health (Bergman, 1990; De Vadder et al., 2014; Jacobson et al., 2018; Schulthess et al., 2019). The SCFA content was compared between treatments, and there were no significant differences in the quantity of SCFAs measured between treatments in the digesta (Supplementary Figure S8) or the faeces (Supplementary Figure S9).

**FIGURE 8:**
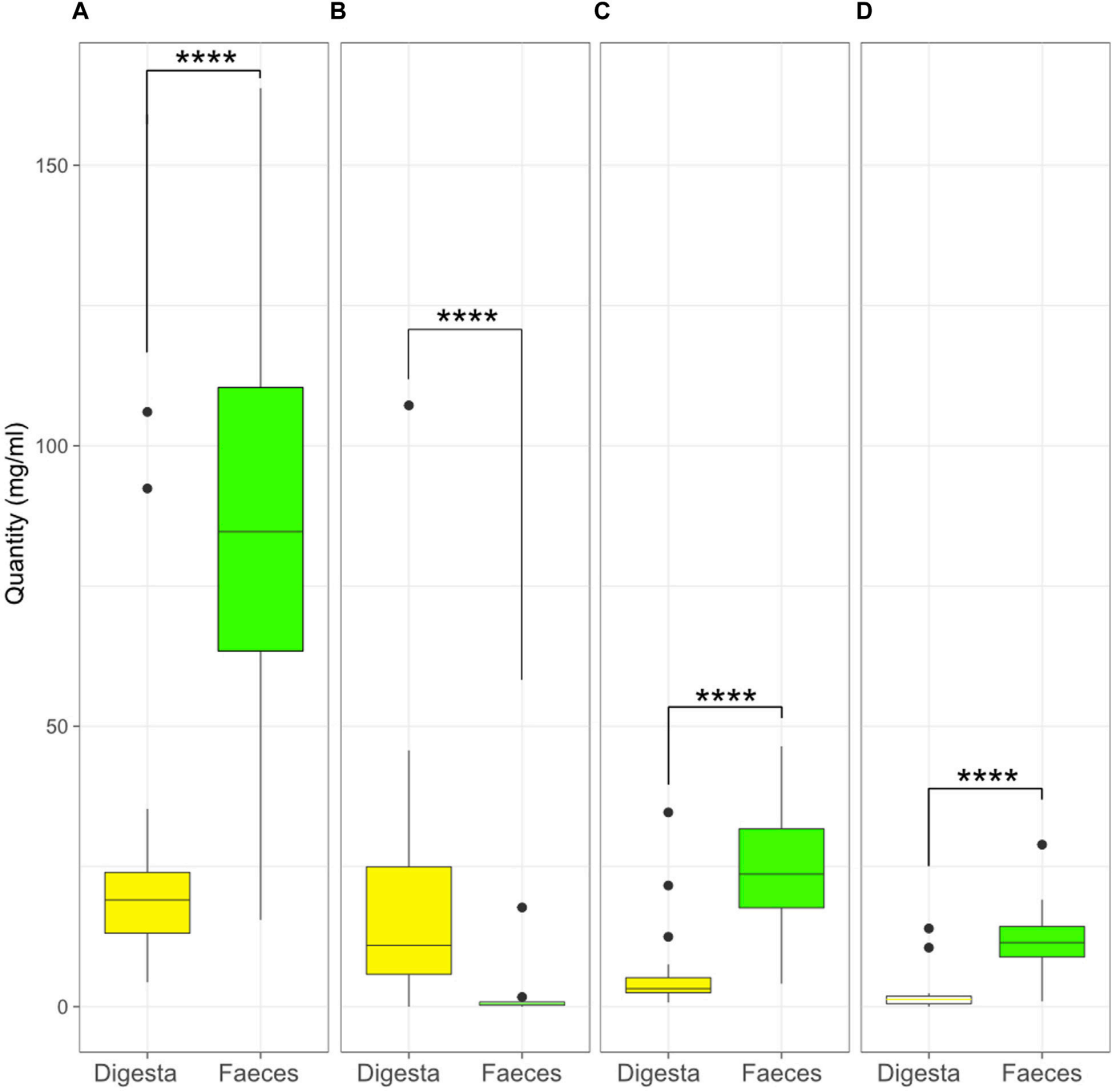
Quantity (mg/mL) of acetic **(A)**, lactic **(B)**, propionic **(C)**, and butyric **(D)** acid in the digesta and the faeces (****, *p* < 0.0001, Kruskal–Wallis test). Box plots show means and quartiles.

The individual SCFA contents were also compared between the digesta and the faeces (Figure 8). The contents of acetic, propionic, and butyric acid were higher in the faeces (*p* < 0.0001) while lactic acid was higher in the digesta (*p* < 0.0001).

**FIGURE 9:**
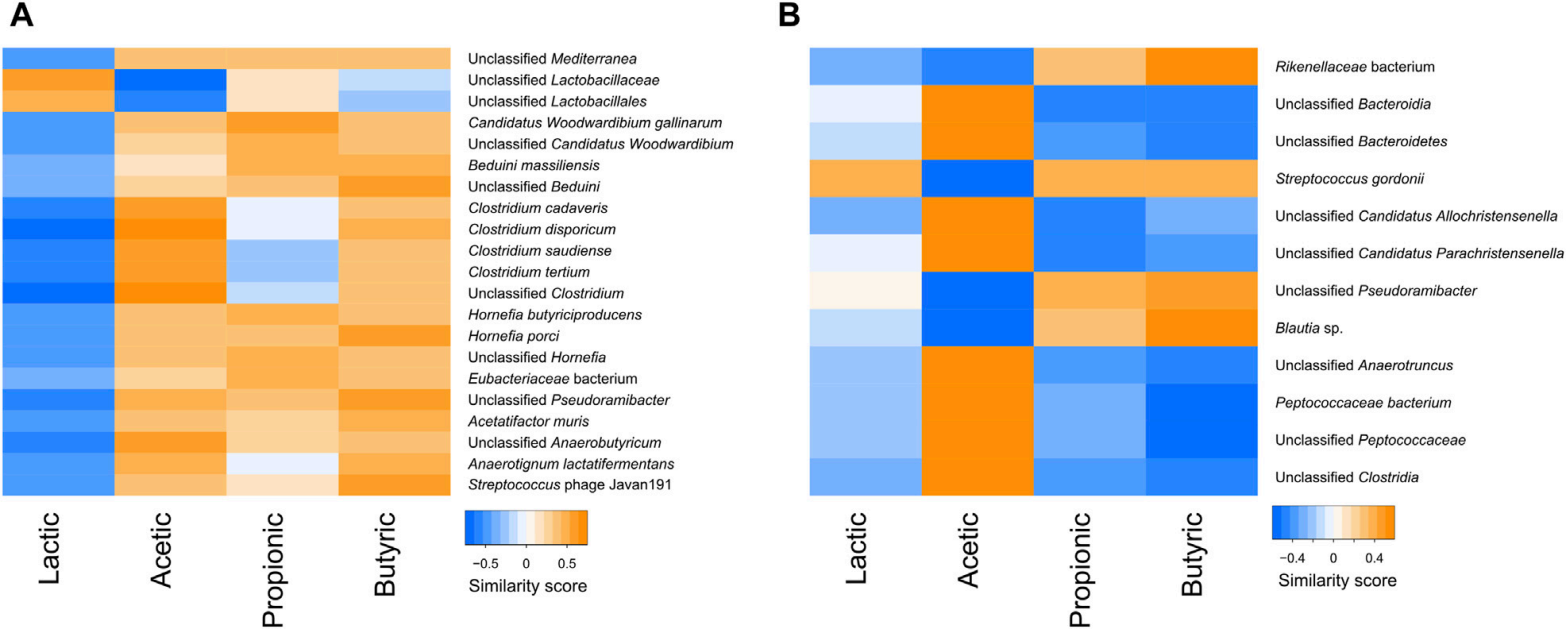
Heatmap of the similarity score between correlated short chain fatty acids and taxa in the digesta **(A)** and the faeces **(B)**.

The correlations between the SCFA content and bacterial species were also tested in the faeces and the digesta. In the digesta, various bacteria, including many *Clostridium* species, were negatively correlated with lactic acid and positively correlated with the other SCFAs. In contrast, lactobacilli were positively correlated with lactic acid, and negatively or not correlated with other SCFAs (Figure 9A). In the faeces, lactic acid did not seem to follow any trend, but there was a clear separation between bacteria that were positively correlated with acetic acid and negatively with butyric and propionic acids, and bacteria that were positively correlated with butyric and propionic acid and negatively correlated with acetic acid (Figure 9B).

## 4 Discussion

The aim of the study was to determine whether the administration of a probiotic (*P. acidilactici*) can mitigate the effects of an antibiotic (tylvalosin) on the microbial community, both taxonomically and functionally, in swine. Although no significant differences in alpha diversity were observed between the treatments, a few tendencies are interesting. The combined treatment samples had the lowest diversity in the faeces, which might be the result of the combined effect of the tylvalosin and the antimicrobial compounds (e.g., bacteriocin and SCFA) produced by the probiotic *P*. *acidilactici*. Both of these antimicrobials target Gram-positive bacteria (Pharmgate, 2021), and *Pediococcus* bacteriocin was shown to target some Gram-negative enterobacteria when the cells were already sub-lethally injured (Osmanagaoglu, 2005). This effect was not observed in the digesta, which could be due to the composition of its microbiota. In fact, the predominant bacterial genus in digesta samples was *Lactobacillus*, and lactobacilli strains were reported to have an inactivating effect on some macrolide antibiotics in chicken microbiota (Devriese and Dutta, 1984). Whether or not this was the case in the present study would need to be investigated specifically with pigs and tylvalosin.

The lack of significance for beta diversity changes between the treatments could partially be explained by the low number of samples. Because there is a high variability between the microbiota of individual pigs, small changes resulting from treatment are not always distinguishable from individual variations (Chen C. et al., 2018). Comparisons between 16S metagenomics data and shotgun sequencing reveal similar conclusions regarding microbial diversity, suggesting that amplicon sequencing alone may suffice to address taxonomic questions.

While the resistome profiles of samples were different between the probiotic treatment and the antibiotic and combined treatments in both the digesta and the faeces, the total ARG score was only higher in the faeces of antibiotic-treated animals. This means that a change in the resistome profile and enrichment of specific genes does not necessarily imply an increase in the total ARG content of a sample. Therefore, simply measuring the quantity of ARGs would be insufficient to properly monitor resistance. There were also fewer increased ARGs in the digesta during antibiotic treatment compared with the faeces, further demonstrating that the digesta and faeces resistomes respond differently to antibiotic treatment. It is important to note that exposure time to in-feed antibiotics could be shorter in the digesta, as the flow of digesta is faster in the ileum than in the colon (Wilfart et al., 2007).

The recuperation period allowed for a decrease in ARGs, although it was lower than the increase occurring during the antibiotic and combined treatments. This means that the microbiome can recover from such treatments, although 3 weeks might not be enough to completely return to an untreated state. Further studies investigating the recovery of the microbiome over a longer period would be necessary to pinpoint the reversal of the treatment effects.

The increase in resolvase and recombinase in the antibiotic-treated faeces samples could suggest higher rates of plasmid transfer. It is not possible to establish what proportion of this difference is explained by higher rates of transfer or by selection of bacteria carrying plasmids with both horizontal transfer genes and ARGs. The fact that two conjugative multi-resistance–carrying plasmids were more frequently detected in samples from the antibiotic treatments, and that Pfam resolvase and recombinase functions were more frequently detected in those samples, indicates a higher risk of horizontal transfer. Other methods, such as epigenomics grouping or culturomics would be necessary to confirm the significance of horizontal transfers between the treatments (Dib et al., 2015). All ARG-carrying plasmids and horizontal gene transfer–related genes that were more present in the antibiotic-treated samples were observed in the faeces, which suggests that the potential for horizontal transfer is higher in the faeces than in the digesta. This represents a problem considering that the faeces are responsible for most of the environmental contamination by pig gut microbiota, notably in fertilized fields (Gao et al., 2020) and pig carcasses (Laforge et al., 2023).

The transferable plasmids that were amplified in the antibiotic and combined treatment samples were originally from *E. coli* and *Klebsiella pneumoniae,* bacteria that were naturally not susceptible to tylvalosin (Pharmgate, 2021); this could explain why the plasmids from *E. coli* and *K. pneumoniae* were more prevalent in antibiotic-treated samples even though they were not carrying macrolide resistance genes. Therefore, naturally non-susceptible bacteria should not be ignored in the selection and transfer of ARGs during resistome studies using antibiotic treatment.

The treatments also did not impact the quantity of SCFAs in the gut microbiome, even for the probiotic-supplemented group; although *P. acidilactici* is known to produce lactic (Klupsaite et al., 2019), butyric, and propionic acid, as well as low levels of acetic acid (Hoseinifar et al., 2017). Analysis of the correlations between SCFAs and bacterial taxa abundance showed that some bacteria were strongly associated with SCFAs, and those correlations varied between digesta and the faeces. The difference in the quantity of SCFAs in the digesta and faeces demonstrate a highly different SCFA profile of the digesta and the faeces, suggesting that the fermentation patterns in the ileum and colon are different in many ways (Urriola and Stein, 2010; Zhang et al., 2018).

In conclusion, the samples collected during antibiotic or combined treatment had very similar profiles of taxonomy, resistance, and plasmidome. This observation would suggest that *P. acidilactici* given simultaneously with tylvalosin does not reduce the resistance selection effect of antibiotic treatment on the resistome. Therefore, there is little to no advantage to this practice. The digesta and faeces responded differently to the treatments in their microbial composition, resistome, and plasmidome; therefore, inferences made from the digesta to faeces, or *vice versa*, are not applicable to the other for microbiome studies. It is important to mention that the pigs in this study were housed separately, and in larger pens there might be a higher transfer of faecal bacteria to the ileum through a faecal-mouth-ileum pathway. Although studies regarding digesta are still important for specific pathogens that colonize the ileum, the faeces seem more important for further investigation of the effects of antibiotics on the pig gut microbiome with respect to meat safety, as faeces are more problematic in term of antibiotics resistance selection, horizontal transfer, and contamination of the food chain. Finally, the present study uses a cannulated pig model to infer the effect of an antibiotic and a probiotic on ileal and fecal microbiota. Although this model is known to enable exploration of the microbiome efficiently (Gao et al., 2018; Lee et al., 2018; Pi et al., 2019), the results should be validated in a commercial setting representing real-life husbandry practices.

## Data Availability

The datasets presented in this study can be found in online repositories. The names of the repository/repositories and accession number(s) can be found in the article/Supplementary Material.
