# Supplementary material for: Effect of a probiotic and an antibiotic on the mobilome of the porcine microbiota
Source: Front Genet. 2024 Mar 28;15:1355134. doi: 10.3389/fgene.2024.1355134 (PMC11006968; doi:10.3389/fgene.2024.1355134)
Supplement: Supplementary file 1 [file DataSheet1.PDF]

Supplementary material

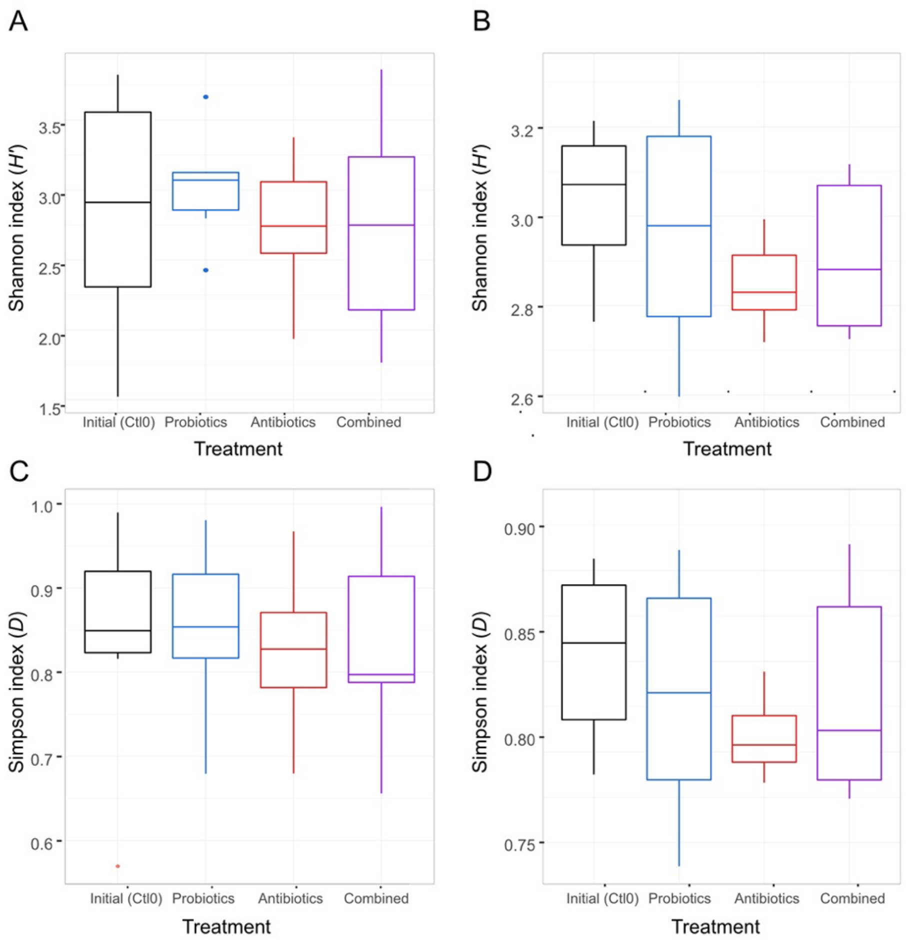

Supplementary Figure 1. Alpha diversity using Shannon index of the digesta (A) and the faeces (B), and alpha diversity of the samples using the Simpson index of the digesta (C) and the faeces (D) for animals treated with the commercial probiotic *Pediococcus acidilactici* MA18/5M (blue), a macrolide antibiotic (tylvalosin, red), or a combination of both (purple). Data from samples taken before the animals received treatments (Ctl0) are shown in black. Box plots show means and quartiles. No comparison was significant ($p > 0.05$, Kruskal-Wallis). Analyses were performed using 16S rRNA gene metagenomic data.

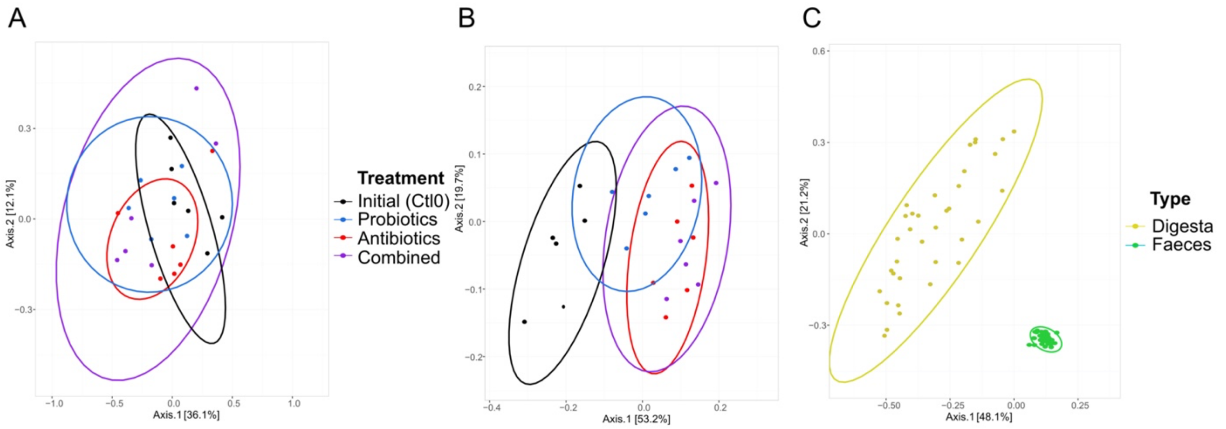

Supplementary Figure 2. Beta diversity by principal coordinate analysis plot using Bray-Curtis dissimilarity of bacterial taxa of the digesta (A), the faeces (B), and all samples (C) using 16S rRNA gene metagenomics for animals treated with the commercial probiotic *Pediococcus acidilactici* MA18/5M (blue), a macrolide antibiotic (tylvalosin, red), or a combination of both (purple). Data from samples taken before the animals received treatments (Ctl0) are shown in black. Each point represents a sample. The distance between points reflects the difference in microbial composition between samples; closer points indicate higher similarity. The principal axes represent dimensions that maximize variance among samples. Percentages indicate the proportion of variance explained by each axis. The ellipses represent a 95% confidence interval.

	Digesta						Faeces					
	Initial	Antibiotics	Probiotics	Combined	Recuperation 1	Recuperation 2	Initial	Antibiotics	Probiotics	Combined	Recuperation 1	Recuperation 2
Lactobacillaceae	51.2	42.4	34.5	48.7	51.8	32.3	1	1.3	1.9	0.9	1.4	1.8
Unclassified *Eubacteriales*	4.4	6	5.2	5.5	4.6	7.6	11.3	19.4	16.6	20.2	15.4	15.5
Prevotellaceae	2.6	2.1	4.3	1.9	4.1	2.8	27	5.4	8.5	5.9	9.6	10.3
Peptostreptococcaceae	8.8	15.6	15.5	11.4	10.3	17.5	0.1	1.3	0.9	1	0.6	1.2
Unclassified	3.5	5.5	3.9	5.9	4.1	4.7	8.7	9.4	9.2	8.7	9.6	8.7
Unclassified *Bacteroidales*	0.8	0.9	2.3	0.8	1	0.8	12.7	10.2	13.4	9.2	12.6	14.3
Unclassified *Firmicutes*	1.4	2.4	2.4	2.2	1.8	2.5	4.2	8.6	6.7	9.6	6.5	6.5
Unclassified Bacteria	0.9	1.5	1.5	1.4	1.2	1.5	5.7	7.7	7.6	7.3	7.6	7.5
Unclassified *Clostridia*	0.5	0.6	0.7	0.7	0.8	0.9	3.2	8.2	6.3	9.1	5.5	5.7
Oscillospiraceae	0.3	0.3	0.4	0.4	0.4	0.3	3.5	4.5	4.2	4.4	3.8	4.2
Turicibacteraceae	1.7	4	3.5	3.1	4	6.8	0	0.2	0.2	0.2	0.1	0.2
Lachnospiraceae	1.1	0.8	1.1	1.2	1.6	1.3	3.2	2.1	2.4	2.3	2.5	2.6
Streptococcaceae	1.5	0.9	8.8	2.1	2	0.5	0.7	0.2	2	0.9	1.8	0.5
Spirochaetaceae	0	0	0.1	0	0	0.1	0.9	3	2.5	2.2	2.9	2.7
Enterobacteriaceae	1.1	3.3	2.3	1.3	1.1	4.5	0.2	0.2	0.1	0.5	0.2	0.1
Bacteroidaceae	1.1	0.8	1.2	0.6	0.6	0.6	1	1.6	1.5	1.2	1.2	1.5
Unclassified *Bacteroidetes*	0.1	0	0.1	0.1	0.1	0.1	1.7	1.4	1.8	1.4	1.5	1.8
Fusobacteriaceae	2.8	0.8	1.4	0.8	1.1	1	0.1	0	0	0	0	0
Unclassified *Bacteroidia*	0	0	0.2	0.1	0.1	0.1	1.7	1	1.3	0.9	1.3	1.1
Remaining taxa (1040)	16.3	11.7	10.6	11.9	9.2	14.2	12.9	14.4	12.9	14.1	15.9	13.7

Supplementary Figure 3. Microbial structure and composition of digesta and faeces samples. Top 20 families based on relative abundance (%) identified. Colour gradients range from blue to orange.

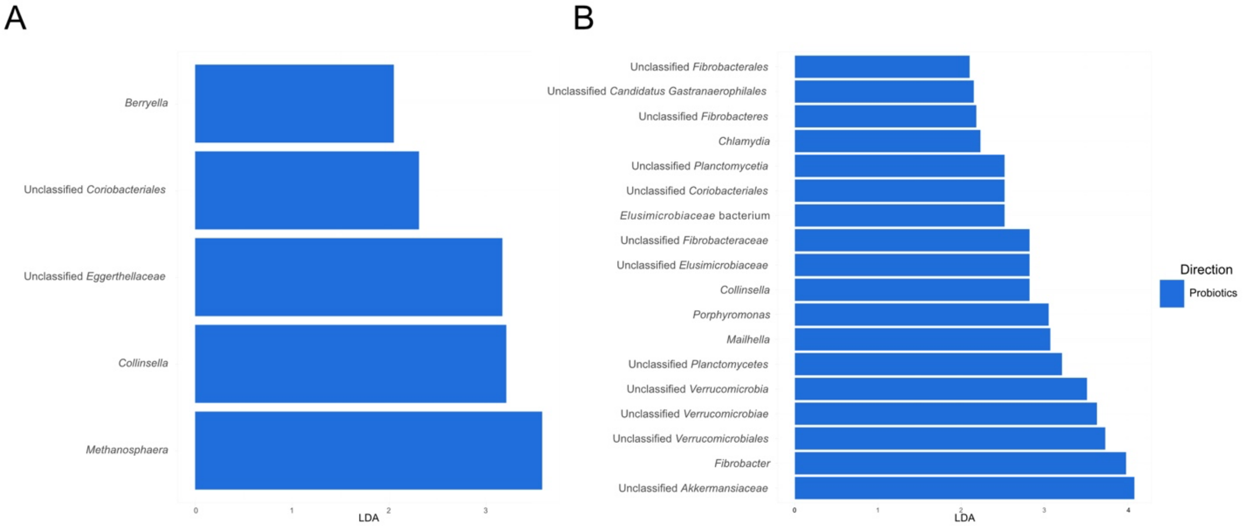

Supplementary Figure 4. Genera that were identified as markers between the antibiotic and probiotic treatment in the digesta (A) and the faeces (B). Only samples from animals fed with the probiotic had markers (score ≥ 2 and a $p < 0.05$).

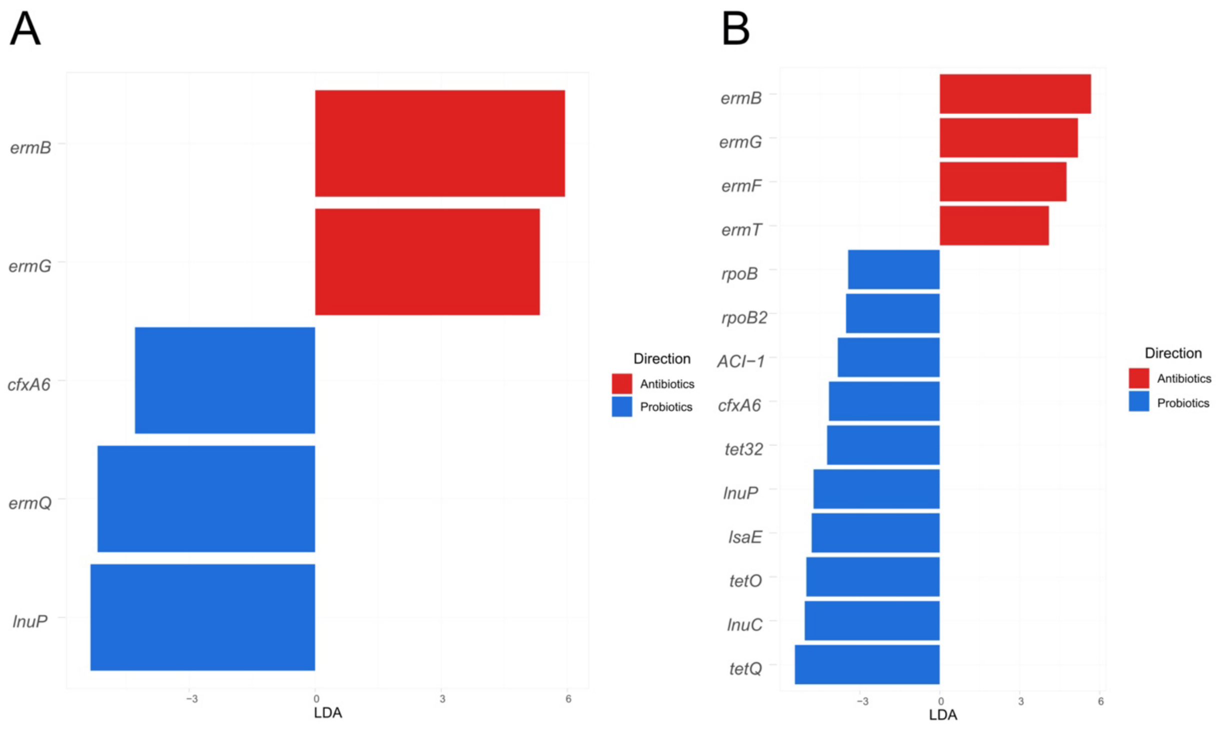

Supplementary Figure 5. Linear discriminant analysis plot of the marker resistance genes (score ≥ 2 and a $p < 0.05$) for the antibiotic and probiotic groups in the digesta (A) and the faeces (B).

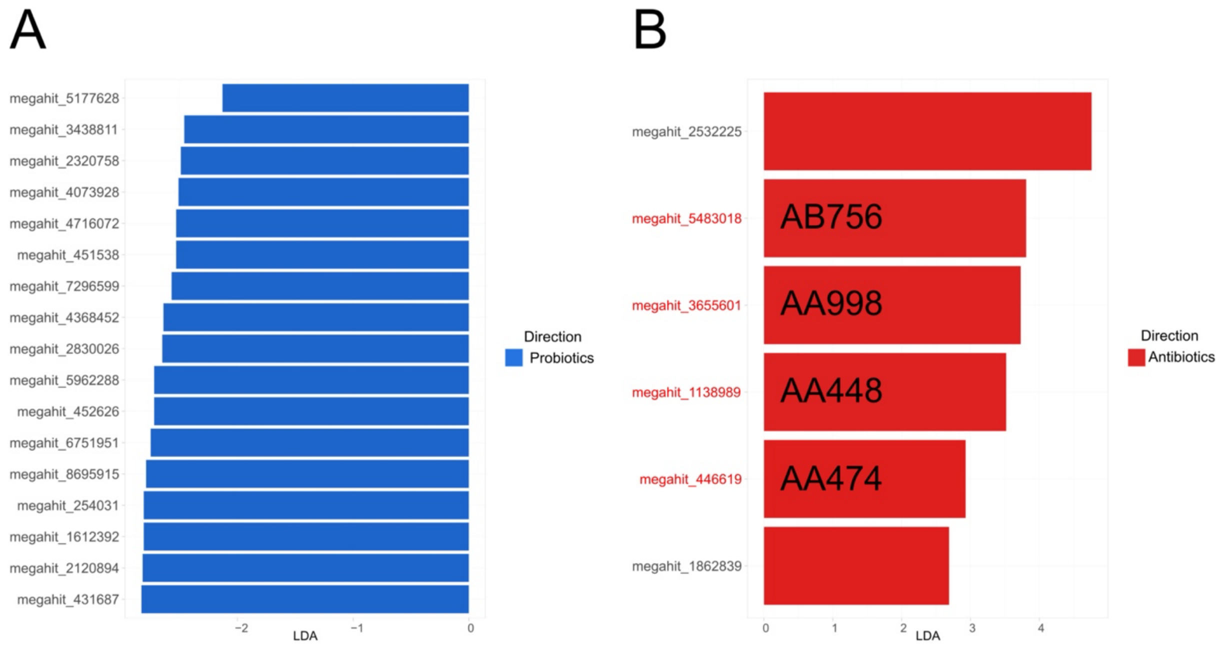

Supplementary Figure 6. Linear discriminant analysis of the marker plasmidic contigs (score ≥ 2 and a $p < 0.05$) found in the digesta (A) and the faeces (B). The names of the contigs in red indicate that they belong to ARG carrying plasmids described in Table 1; the names of the plasmids are written in the bar.

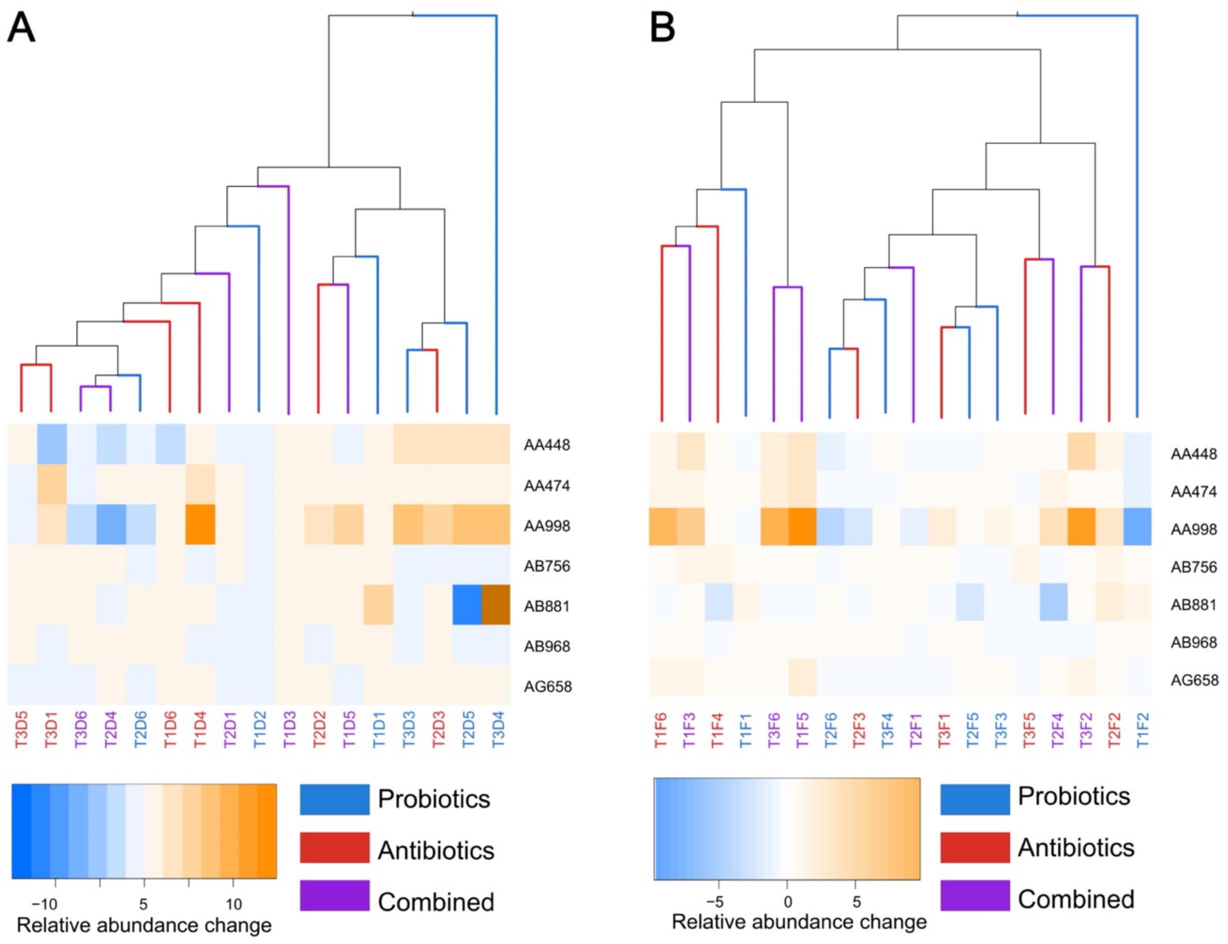

Supplementary Figure 7. Hierarchical clustering of the plasmidome profiles and heatmap of the change in relative abundance of antibiotic resistance gene–carrying plasmids between treatment periods and their preceding recuperation periods in the digesta (A) and the faeces (B). The sample names correspond to the treatment period of the samples: T1, T2, or T3; the nature of the samples: faeces (F) or digesta (D); and the pig from which the sample was taken: pig 1 to 6. Blue corresponds to probiotic treatment, red to antibiotic treatment, and purple to combined treatments.

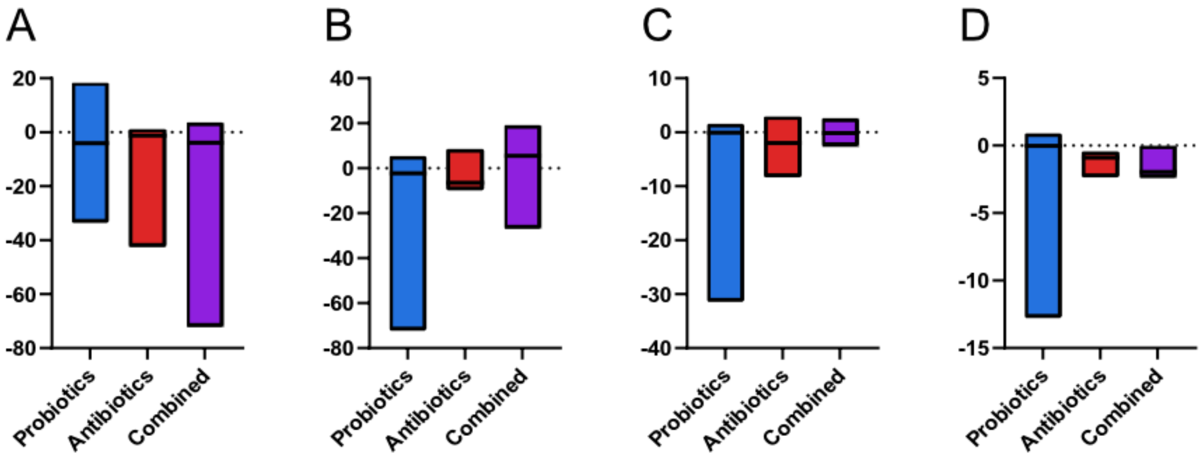

Supplementary Figure 8. Change in the quantity of lactic (A), acetic (B), propionic (C), and butyric (D) acid in digesta samples for animals treated with the commercial probiotic *Pediococcus acidilactici* MA18/5M (blue), a macrolide antibiotic (tylvalosin, red), or a combination of both (purple). No comparison was significant ($p > 0.05$, Kruskal-Wallis).

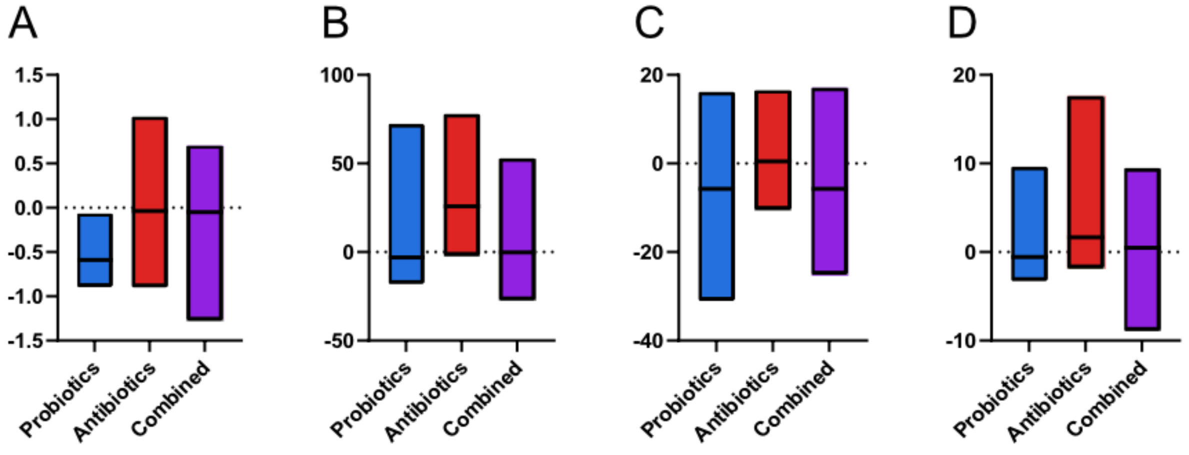

Supplementary Figure 9. Change in the quantity of lactic (A), acetic (B), propionic (C), and butyric (D) acid in faeces samples for animals treated with the commercial probiotic *Pediococcus acidilactici* MA18/5M (blue), a macrolide antibiotic (tylvalosin, red), or a combination of both (purple). No comparison was significant ($p > 0.05$, Kruskal-Wallis).
